# Dynamics of SARS-CoV-2 Spike-IgG throughout Three COVID-19 Vaccination Regimens: A 21-Month Longitudinal Study of 82 Norwegian Healthcare Workers

**DOI:** 10.3390/v15030619

**Published:** 2023-02-23

**Authors:** Marita Helen Augustinussen, Garth D. Tylden, Christine Hanssen Rinaldo

**Affiliations:** 1 Department of Microbiology and Infection Control, University Hospital of North Norway, N-9038 Tromsø, Norway; 2 Department of Medical Biology, UiT The Arctic University of Norway, N-9037 Tromsø, Norway; 3 Metabolic and Renal Research Group, Department of Clinical Medicine, UiT The Arctic University of Norway, N-9037 Tromsø, Norway

**Keywords:** BNT162b2, ChAdOx1 nCoV-19, Liaison S1/S2-IgG, SARS-CoV-2 serology, antibody decline, spike

## Abstract

To facilitate interpretation of clinical SARS-CoV-2 anti-spike IgG analyses post-vaccination, 82 healthcare workers were followed through three vaccination-regimens: two regimens were comprised of two doses of BNT162b2 three or six weeks apart, followed by a dose of mRNA-vaccine, and in the other regimen, the first dose was replaced by ChAdOx1 nCov-19. After each dose, anti-spike IgG was compared between regimens. As many participants became infected, anti-spike IgG persistence was compared between infected and uninfected participants. Thirteen to twenty-one days after the first dose, seroconversion, and the median anti-spike IgG level in the ChAdOx1 group was significantly lower than in the BNT162b2 groups (23 versus 68 and 73 AU/mL). The second dose caused a significant increase in anti-spike IgG, but the median level was lower in the BNT162b2-short-interval group (280 AU/mL), compared to the BNT162b2-long-interval (1075 AU/mL) and ChAdOx1 (1160 AU/mL) group. After the third dose, all groups showed increases to similar anti-spike IgG levels (2075–2390 AU/mL). Over the next half year, anti-spike IgG levels declined significantly in all groups, but appeared to persist longer after post-vaccination infection. This is the first three-dose study with one dose of ChAdOx1. Despite initial differences, all vaccine regimens gave similarly high antibody levels and persistence after the third dose.

## 1. Introduction

Since the emergence of severe acute respiratory syndrome coronavirus-2 (SARS-CoV-2) in December 2019, more than 635 million infections and 6.3 million deaths have been reported [1], though the true number of deaths is likely 3–4 times higher [2]. 

In Europe, the messenger ribonucleic acid (mRNA) -based vaccine BNT162b2 produced by Pfizer-BioNTech and mRNA-1273 produced by Moderna, were authorised for use on the 21 December 2020 and 6 January 2021, respectively, while the recombinant adenovirus-based vaccine ChAdOx1 nCov-19 (ChAdOx1) produced by Oxford-AstraZeneca was authorised on the 29 January 2021 [3]. These three vaccines were all found to offer good protection against severe coronavirus disease 2019 (COVID-19), hospitalisation, and death [4,5,6]. Without the rapid roll out of these vaccines, the number of deaths in Europe would likely have been much higher.

In Norway, the healthcare workers were the first to receive COVID-19 vaccines. From January 2021 the mRNA-1273 and BNT162b2 were offered, followed in March 2021 by ChAdOx1. All three vaccines deliver a nucleic acid encoding the SARS-CoV-2 spike (S) glycoprotein which is then produced in vivo, eliciting a spike-specific immune response. Originally, a two-dose regimen of each of these vaccines was planned, but due to severe side effects and death of some healthcare workers receiving ChAdOx1 [7], this vaccine was withdrawn from the market in Norway. As a second dose, these healthcare workers instead received BNT162b2. Later, it was revealed that the adenoviral component of ChAdOx1 could cause a vaccine-induced immune thrombotic thrombocytopenia via formation of antigenic complexes with platelet factor 4 [7,8,9]. Due to reports of rapidly declining protection against infection and disease, a third dose was tested and recommended [10,11].

In order to detect SARS-CoV-2 antibodies and to distinguish convalescent or previous SARS-CoV-2 infection from COVID-19 vaccination, our diagnostic laboratory established one assay targeting anti-S immunoglobulin G (IgG) and one assay targeting anti-nucleocapsid (N) IgG. The anti-S IgG assay was expected to detect antibodies generated in response to both SARS-CoV-2 infection and vaccination and the anti-N IgG assay was expected to only detect antibodies generated in response to SARS-CoV-2 infection. However, when vaccination roll-out started, there were no studies evaluating the performance of our chosen tests in vaccinated populations.

To address the lack of data relevant to our routine clinical serology, we set out to study the dynamics of the anti-S IgG response after vaccination in SARS-CoV-2 naive healthcare workers. Due to a combination of public policy and vaccine supply issues, three different vaccination regimens were compared.

## 2. Materials and Methods

### 2.1. Study Design, Participants and Groups

**Figure 1:**
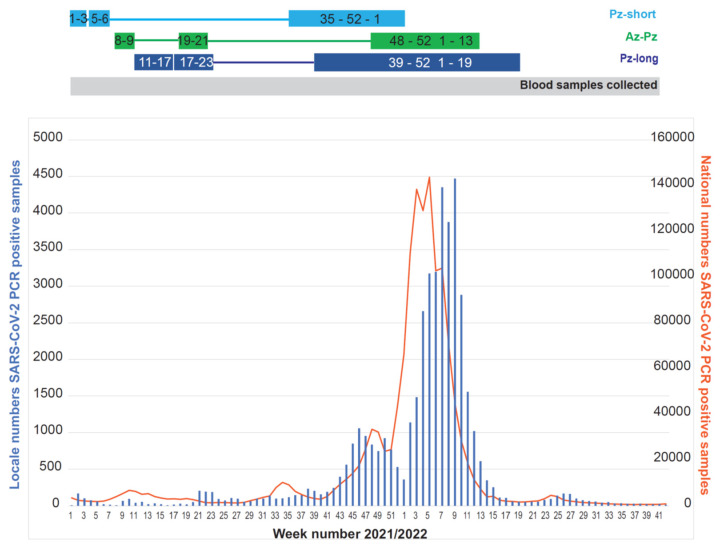
Timeline of the study. Upper part: time of vaccination. The Pz-short (light blue), Az-Pz (green) and Pz-long (dark blue) groups received three vaccine doses at different timepoints and intervals, illustrated by boxes with week numbers. Blood samples were collected from 1 January 2021 until 17 October 2022, i.e., week 1 of 2021 up to and including week 41 of 2022. Lower part: pandemic curve. Numbers of polymerase chain reaction (PCR) confirmed SARS-CoV-2 infections from our laboratory (blue bars) and total national numbers (orange graph) during the time of our study. At the beginning of the study, numbers of diagnosed infections were low but peaked during the period in which the third dose was administered.

Eighty-three hospital staff aged between 21 and 65 years working in the Department of Microbiology and Infection Control at the University Hospital of North Norway in Tromsø, Norway self-recruited to the study that was performed from 1 January 2021 to 17 October 2022 (Table 1), a time interval with varying infection rates (Figure 1). Later, one person withdrew consent and was excluded. During the course of the national vaccination program, participants received COVID-19 vaccines independently of their participation in our study and were grouped according to the vaccine-regimen given. The interval between the first two doses and the vaccine type that was given varied through the study period and gave rise to three different vaccine groups.

The vaccine groups were named after the producer of the first vaccine dose and either the producer of the second dose or the length of the interval between the first two doses (Figure 1, Table 1). The Pfizer-BioNTech (Pz)-short group: 45 participants received BNT162b2 for the first two doses with a short interval, except for one participant who received the mRNA-1273 as the second dose. The AstraZeneca-Pfizer-BioNTech (Az-Pz) group: 21 participants received ChAdOx1 as the first dose and BNT162b2 as the second dose. The Pfizer-BioNTech (Pz)-long group: 16 participants received BNT162b2 as the first two doses with a long interval. All groups received either BNT162b2 or mRNA-1273 as the third dose.

SARS-CoV-2 infection was reported by participants either based on a self-performed positive rapid antigen test or a positive polymerase chain reaction (PCR) test performed in our laboratory. Testing was initiated by the participants themselves. In addition, unreported infections were detected through analysis of all serum samples for anti-N IgG.

### 2.2. Serum Samples and Analyses

Serum samples were tested for SARS-CoV-2 anti-S IgG and anti-N IgG at intervals approximating three weeks after the first vaccine dose and three weeks, three months, and half a year after the second and third dose, as well as after SARS-CoV-2 infection. 

Some participants tested more than one sample during a given interval and some tested additional samples outside the intervals. In total, 626 serum samples were tested. The concentration of anti-S IgG was measured with Liaison SARS-CoV-2 S1/S2 IgG assay (DiaSorin), and the concentration of anti-N IgG was measured with Abbott SARS-CoV-2 IgG assay (ARCHITECT and Alinity system). The anti-S IgG assay is a two-step chemiluminescent microparticle immunoassay with recombinant S1- and S2-antigen-coated microparticles and isoluminol-conjugated anti-human IgG. Antibody concentrations are reported in arbitrary units (AU/mL), with a range of 3.8 to 400 AU/mL. Positive results were defined as ≥15 AU/mL. An automatic 1:10 dilution and reanalysis was performed for samples with a result >400 AU/mL. The anti-N IgG assay is also a two-step chemiluminescent microparticle immunoassay, but this assay has recombinant nucleocapsid protein-coated microparticles and acridinium-labelled anti-human IgG. The concentration of anti-N IgG is measured in relative light units (RLU), with cut-off defined as Index 1.4. The Abbott SARS-CoV-2 IgG assay was initially run on the ARCHITECT system, and after the 1st of December 2021 on the Alinity system due to system upgrade in the laboratory. All analysis was performed according to the manufacturers’ instructions.

### 2.3. Statistical Analyses

The primary outcome was anti-S IgG levels at three weeks after the first dose and at three weeks, three months, and half a year after the second and third doses in the different vaccine groups. As most of the participants contracted the SARS-CoV-2 infection late in the study period, we decided to compare the persistence of the anti-S IgG levels after two and three vaccine doses, and after post-vaccination SARS-CoV-2 infection as a secondary outcome. After detection of infection in a participant, subsequent samples from that participant were removed from vaccine-group comparisons. Serum samples taken in the following time intervals were included in the statistical analysis. First dose: 13–21 days. Second and third dose, and after infection: 13–31 days, 80–120 days, and 170–220 days, respectively. The comparison of anti-S IgG concentrations between vaccine groups was conducted by the Wilcoxon rank sum test and the Kruskal–Wallis test at every time interval. A Wilcoxon rank sum test with continuity correction was performed to compare the median antibody levels over time in the same group. When multiple data were available within a time interval, a mean was calculated and coded as one observation in the Wilcoxon rank sum tests and Kruskal–Wallis tests. *p*-values less than 5% were considered statistically significant. The vaccine groups were merged, and the linear mixed model regression “Imer” was used to compare mean changes in antibody levels over time after a second vaccine dose, after a third vaccine dose, and after a confirmed SARS-CoV-2-infection. Statistical significance was determined with a 95% confidence interval (CI) not including 0 and not overlapping. All statistical analyses were performed using R, version 4.1.1 (Vienna, Austria).

## 3. Results

### 3.1. Characterization of the Cohort and Serum Samples 

The cohort had a mean age of 44 years and was dominated by female participants (Table 1). All participants received two vaccine doses and 88% received three doses. The mean interval between the first and second dose varied from 22 days in the Pz-short group to 39 days in the Pz-long group, and 79 days in the Az-Pz group (Figure 1, Table 1). The distribution of serum samples within each time interval was similar between the three vaccine groups (Figure S1).

### 3.2. The Anti-S IgG Response Elicited by the First, Second and Third Vaccine Dose

As our study started shortly after the first COVID-19 vaccine was authorised, little information was available on the dynamics of the IgG response to COVID-19 vaccines. However, studies on SARS-CoV-2 infection in humans had shown that antibody levels peaked within three to seven weeks post-symptom onset and declined from eight weeks post-symptom onset [12,13]. Additionally, our initial investigations of a few subjects that had been vaccinated with BNT162b2 showed seroconversion by 14 days after the first vaccine dose. In order to study the anti-S IgG response elicited by vaccination, we therefore aimed to perform testing about three weeks after each vaccine dose, which for the Pz-short group was the longest interval attainable between the first two doses. Notably, after only one vaccine dose, the Pz-short and the Pz-long group had received the same treatment. 

**Figure 2:**
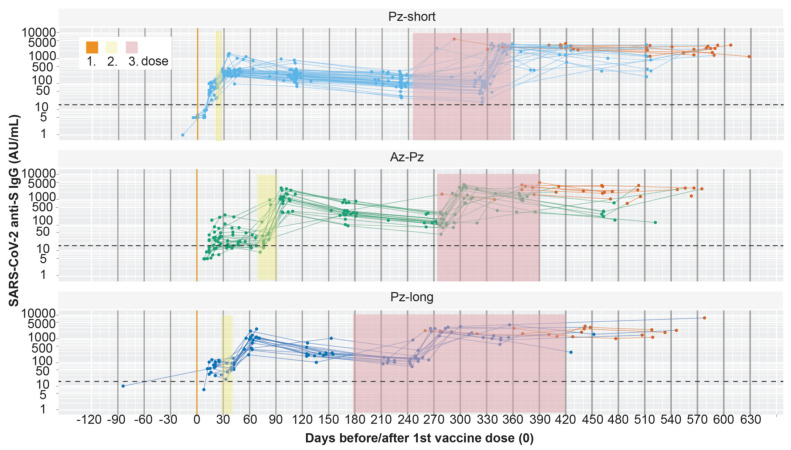
The dynamics of anti-S IgG following vaccination: timeline of individual participants. Anti-S IgG (AU/mL) was measured in individual participants over time and normalised at the first vaccine dose (vertical orange line). The assay positive threshold is marked with a dashed line. Each participant is represented by a line joining their measured values (light blue, green, and dark blue dots). Red dots indicate that the serum sample is analysed after a reported infection based on either a self-performed positive rapid antigen test, a positive PCR test, or detection of anti-N IgG. The time interval for the second and the third dose is shown in yellow and pink, respectively. Between 13 and 21 days post vaccination, seroconversion is detected in all participants in the Pz-short and Pz-long group, but not in the Az-Pz group. The anti-S IgG response over time, normalised at the second dose, is shown in Figure S2.

**Figure 3:**
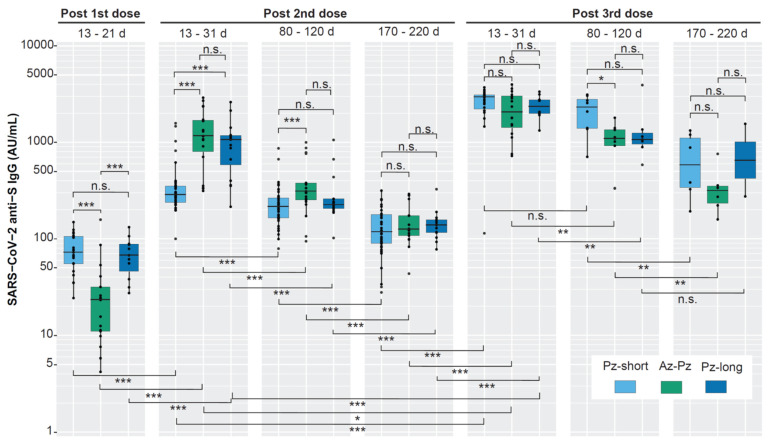
Anti-S IgG levels (AU/mL) elicited by the first, second, and third vaccine dose in the three vaccine groups and anti-S IgG persistence up to 21 months post initial vaccination. For the 1st dose, only one time period (13–21 days post-vaccination) was included. For the second and third doses, two additional time periods (80–120 days and 170–220 days post vaccination) were included. The box denotes the interquartile range (IQR), the horizontal line inside the box denotes median. The vertical whiskers that extend from the box indicate 1.5 × IQR. The Wilcoxon rank sum test was used to compare median antibody levels between groups at every time interval (shown above the boxes), and over time within the same group (shown below the boxes, Table 2). A Kruskal–Wallis test was also used to check if at least one of the three vaccine groups were significantly different from the others at the same time interval (Table S1). *p*-value < 0.05 is noted *, *p* < 0.01 **, and *p* < 0.001 ***, and non-significant (n.s.).

About three weeks (13–21 days) after the first vaccine dose, seroconversion could be detected in all participants in the Pz-short and Pz-long group with samples during that time interval, i.e., in 21 and 11 participants, respectively (Figure 2). In contrast, only 10 of 17 (59%) participants of the Az-Pz group had seroconverted. In increasing order, the median anti-S IgG levels of the groups were 23, 68, and 73 AU/mL, for the Az-Pz-, Pz-short-, and the Pz-long group, respectively (Figure 3, Table 2). The Az-Pz group had a significantly lower antibody level than the other groups (Table S1). There was no significant difference between the Pz-short and Pz-long groups, supported a resembling composition of the two Pz-groups.

About three weeks (13–31 days) after the second vaccine dose, all participants had seroconverted (Figure S2) and the median anti-S IgG level of all vaccine groups was significantly increased (Figure 3, Table 2). The Az-Pz group showed a 50-fold increase, the Pz-long group a 16-fold increase, and the Pz-short group an only 3.9-fold increase. In increasing order, the median anti-S IgG levels were 280, 1075, and 1160 AU/mL, for the Pz-short-, the Pz-long, and the Az-Pz-group, respectively. This time, the Pz-short group had a significantly lower antibody level compared to the other groups, while there was no significant difference between the Az-Pz and the Pz-long groups (Table S1).

About three weeks (13–31 days) after the third dose, the median anti-S IgG levels were significantly increased compared to three weeks after the second dose for all groups (Figure 3, Table 2). The highest increase, 10.4-fold, was found in the Pz-short group, followed by a 2.2- and 1.8-fold increase in the Pz-long and Az-Pz group, respectively. In increasing order, the median anti-S IgG levels of the Az-Pz, Pz-long, and the Pz-short group were 2075, 2390, and 2920 AU/mL, respectively. This time, the median antibody levels were not significantly different between any vaccine groups. (Table S1). It is notable that no SARS-CoV-2 infection was detected before the second dose (Figure S2).

In summary, all three vaccine regimens elicited an anti-S IgG response to the first, second, and third dose. However, for some participants in the Az-Pz group, seroconversion was first seen after the second dose. The median antibody level significantly increased with an increasing number of doses and was highest after the third dose. The antibody response elicited by the initial ChAdOx1 dose was significantly lower than the response elicited by the first BNT162b2 dose, while the second BNT162b2 dose elicited a significantly lower median antibody level in the Pz-short group compared to the other two groups. However, three weeks after the third dose, there were no significant differences between the anti-S IgG levels of the three groups. 

### 3.3. The Persistence of the Anti-S IgG after Vaccination

At three months (80–120 days) after the second dose, a significant decline in anti-S IgG was seen for all vaccine groups (Figure 3, Table 2). A 1.3-fold, 3.7-fold, and 4.6-fold reduction was seen the Pz-short group, and the Az-Pz- and the Pz-long group, respectively. The Pz-short group had a significantly lower anti-S IgG level compared with the Az-Pz group, but no significant difference was seen between the two Pfizer groups or between the Az-Pz and Pz-long group. Half a year (170–220 days) after the second dose, additional significant declines were observed for all three groups. A 1.8-fold, 2.5-fold, and 1.7-fold reduction was seen in the Pz-short, the Az-Pz, and the Pz-long groups, respectively. At this point, there was no significant difference in the anti-S IgG levels between the groups.

As described above, the highest anti-S IgG levels were detected about three weeks after the third dose for all three vaccine groups. Three months (80–120 days) later, significant declines were observed for the Az-Pz group (1.9-fold) and the Pz-long group (2.2-fold) but not for the Pz-short group (Figure 3, Table 2). At this time, the Pz-short group had a significantly higher antibody level than the Az-Pz-group. Half a year (170–220 days) after the third dose, significant declines were observed for the Pz-short and the Az-Pz groups but not for the Pz-long group. A 3.7-fold and 3.6-fold reduction was seen in the Pz-short and the Az-Pz group, respectively. There were no significant differences in the anti-S IgG levels between the groups. It should be noted that the number of participants contributing with serum samples at three months and half a year after the third vaccine dose was low.

In summary, anti-S IgG was detectable in all groups three months and then half a year after the second and third vaccine dose, but the anti-S IgG level decreased significantly over time. There were only two exceptions to this, both observed after the third dose: a relatively stable antibody level in the Pz-short group between three weeks and three months, and in the Pz-long group between three months and half a year. Importantly, half a year after the second and third dose, differences in anti-S IgG level between the groups appeared to even out. 

### 3.4. SARS-CoV-2 Infections 

At inclusion time, there was a very low number of SARS-CoV-2 infections diagnosed in our local diagnostic laboratory as well as in Norway (Figure 1), and none of the participants had been diagnosed with infection or had detectable levels of anti-N IgG. During this time, the SARS-CoV-2 Alpha variant dominated in Norway [14]. At the turn of the year 2021 to 2022, the number of diagnosed SARS-CoV-2 infections rapidly increased, coinciding with the introduction of the Delta- and subsequently the Omicron variants in Norway [15] (Figure 1). In total, 61 participants (74%) reported infection confirmed either by PCR or a self-performed rapid antigen test (Figure 2, Table 3). Not all of these delivered a serum sample for analysis after infection, but for 37 participants (61%), one or more serum samples were analysed and 27 (73%) had detectable anti-N IgG. In addition, anti-N-IgG was detected in five participants without reported infection, considered asymptomatically infected. In total, infection was detected in 66 (80%) of the participants during the study period. For participants with detectable anti-N IgG, the samples were taken between day 12 to 201 post-diagnosis (median 46), and for participants without detectable anti-N IgG, the samples were taken between day 5 to 292 post-diagnosis (median 93). For the majority of the infected participants, infection occurred after the third dose.

In summary, infection was detected in 66 participants (80%), and five of them were retrospectively diagnosed based on anti-N IgG. Only 73% of the examined participants with a reported infection had detectable anti-N IgG. Before day 12 and after half a year (201 days) post-diagnosis, anti-N IgG was not detected. 

### 3.5. The Persistence of Anti-S IgG after Post-Vaccination Infection

**Figure 4:**
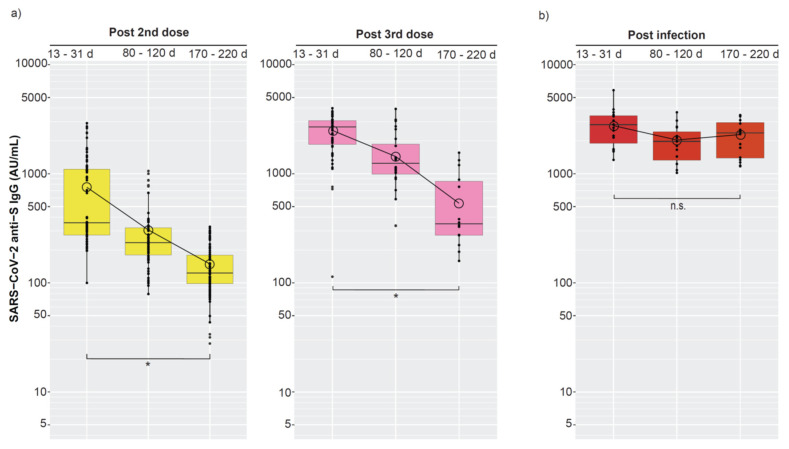
Decline of anti-S IgG in vaccinated individuals and individuals with a hybrid immunity caused by SARS-CoV-2 infection after vaccination. At the indicated time points (after the second and third vaccine dose and after confirmed infection), anti-S IgG (AU/mL) levels were merged for the three vaccine groups and a linear mixed model regression was used to compare the changes in mean anti-S IgG levels. The box denotes IQR, the line inside the box denotes median, whiskers that extend from the box indicate 1.5 × IQR. The additional “O”-circle represents the mean which is extracted from linear mixed regression models in which we adjusted for repeat measurements per participant. Similarly, the lines between the circles illustrate the slope between each interval. Significance is denoted * and non-significant (n.s.). Detailed data in Table 4. (**a**) Antibody level decline after two and three vaccine doses, (**b**) Antibody level decline after additional infection.

Due to the low number of samples from participants after infection, the vaccine groups were merged before further analysis. A regression model was used to investigate the decline of anti-S IgG after the second and third vaccine dose, and after vaccination followed by infection. The merged data showed a significant decline in mean anti-S IgG levels over time post-vaccination (Figure 4a, Table 4) in line with the previously described decline in median anti-S IgG levels found for each of the separate vaccine groups (Figure 3). In more detail, from three weeks to half-a year after the second vaccine dose, a significant 5-fold decline was observed in mean anti-S IgG level. Similarly, after the third vaccine dose a significant 4.6-fold decline was observed.

For infected participants, no significant decline in the mean anti-S IgG level was observed from three weeks to half a year after infection (Figure 4b, Table 4), but the 95% CI for the slope of the post-vaccination infection group was wide and overlapped the 95% CI from the slopes of the post-vaccination (uninfected) group. Finally, we noted that the mean anti-S IgG level three weeks post infection (M = 2760 AU/mL) (Table 4), is higher than anti-S IgG levels measured in our laboratory in infected non-vaccinated individuals, as levels above 400 AU/mL are seldom seen (results not shown).

In summary, a similar fold-reduction in the mean anti-S IgG after the second- and the third vaccine dose was found. On the contrary, no decline of anti-S IgG following post-vaccination SARS-CoV-2 infection was demonstrated. 

## 4. Discussion

In this observational study examining the dynamics of anti-S IgG in three different vaccine regimens, we found that all three regimens elicited an anti-S IgG response, and that the median antibody level significantly increased with an increasing number of doses. The response elicited by the initial dose of ChAdOx1 vaccine was significantly lower than that elicited by the first dose of BNT162b2, and a lower proportion of participants in the Az-Pz group demonstrated seroconversion 13 to 21 days post vaccination. All groups received BNT162b2 as the second dose, but the response elicited was significantly lower in the short dosing-interval group (Pz-short). However, after the third dose, anti-S IgG rose to similar, high levels in all groups. Over the next half year, significant declines were observed in all vaccine groups, and differences in anti-S IgG level between the groups evened out. Decline of anti-S IgG was not demonstrated after post-vaccination infection, however, the slopes were not significantly different.

Our finding of a significantly lower anti-S IgG level after one dose of ChAdOx1 vaccine compared to one dose of BNT162b2 is consistent with earlier published data. After a single dose, the ChAdOx1 vaccine elicits a weaker IgG response than the BNT162b2 vaccine, and, in contrast, the T cell response seems to be stronger for the former [16,17]. About three weeks post-vaccination, we also observed that a lower proportion of individuals in the Az-Pz group had seroconverted, which may suggest that the humoral response to this vaccine takes more time than for the mRNA-based vaccines. We can therefore not exclude that the 13 to 21-day interval used was too short to detect peak levels of anti-S IgG in the Az-Pz group. In support of this, a study by Falsey and colleagues reported a continued increase in anti-S IgG and neutralising antibodies 28 days post ChAdOx1 vaccination [6]. 

After the second dose, which for all groups was the BNT162b2 vaccine, the strongest increase in anti-S IgG was found in the Az-Pz group while the weakest increase and lowest antibody level was found in the Pz-short group. Since the only difference in the vaccination scheme of the Pz-short and the Pz-long group was the interval between the first and second dose (22 days and 39 days, respectively), our results suggest that the period between the third and sixth week after the first dose is important for the development of a strong humoral response to the second dose. An extended interval between the first and the second dose of BNT162b2 vaccine has already been recognised by others to enhance the peak antibody response [18]. A study by Pozzetto et al. [16] shows that a four-week interval between the first and second dose may be sufficient to obtain similar levels of anti-S1 IgG four weeks after homologous vaccination with BNT162b2 and heterologous vaccination with BNT162b2 and ChAdOx1. The largest increase in anti-S IgG being found in the Az-Pz group may be explained by the heterologous prime-boost schedule, previously demonstrated by the UK COMCOV trial to be highly efficient [19]. Additionally, the long interval of 10–13 weeks between the first and the second dose in the Az-Pz group may have influenced the result, as lengthening of the interval between the first and the second dose from four to twelve weeks is found to enhance the humoral response both in homologous and heterologous schedules [20]. 

After the third dose, the anti-S IgG levels rose significantly, and notably, there were no more differences in the anti-S IgG level between the three groups. The ability of a third dose of BNT162b2 or mRNA-1273 to increase humoral and cellular immunity and protection against symptomatic infection has been demonstrated by multiple studies [21,22]. In spite of this, we have not found other studies finding similar levels of anti-S IgG after three-dose regimens with a single initial dose of ChAdOx1. In contrast, the COV-BOOST trial showed that heterologous boosting with an mRNA vaccine as the third dose was superior to homologous boosting when the ChAdOx1 vaccine was initially used [22]. Moreover, homologous three-dose BNT162b2 schedules showed higher anti-S IgG at 28 and 84 days after the third dose, compared to heterologous schedules with two initial doses of ChAdOx1 [23]. As far as we can see, other published three-dose studies include two doses of the ChAdOx1 vaccine before a third dose with an mRNA vaccine. The uneven distribution of the mRNA-1273 vaccine as the third dose may have influenced our results, as mRNA-1273, due to the 3.3 times higher RNA content, has been found to be more immunogenic than BNT162b2 [24]. 

The decline in the COVID-19 vaccine’s induced antibodies over time has been demonstrated in several studies [11,25,26,27]. In agreement with this, at three months and half a year after the second and third vaccine dose, we observed significant declines in anti-S IgG levels. There were only two exceptions to this, both observed after the third dose. These exceptions may be due to the low number of samples after the third dose or other factors such as the variation in interval between the second and third dose, the uneven distribution of mRNA-1273, or undetected infections. 

During our study, we detected SARS-CoV-2 infection in 66 participants, and 37 of them continued giving serum samples after confirmed infection. Anti-N IgG was found in 27 (73%) of these participants, but half a year after infection, no anti-N IgG was detected. In a study by Muecksch and colleagues [28], the sensitivity of the Abbott SARS-CoV-2 anti-N IgG assay was found to decline with time to only 71% at 81 days after diagnosis, compared to 98% at 21 to 40 days and 85–91% at 41 to 80 days after diagnosis. Our findings are in line with these. A steep decay of anti-N IgG has also been found with other assays [29]. Since a negative anti-N IgG result cannot rule out previous SARS-CoV-2 infection, other methods are needed. Time- and dose-dependent reference levels for anti-S IgG, such as we have shown in this study, can facilitate interpretation of clinical anti-S IgG results when the patients’ detailed vaccination histories are available. 

Anti-S IgG appeared to persist at a higher level in post-vaccination infected individuals, though the finding was not significant, possibly due to low sample size. Several studies on the durability of humoral response after SARS-CoV-2 infection have demonstrated that both binding and neutralising antibody levels are only modestly decreased at 8 to 10 months after the infection [30,31]. In many individuals, SARS-CoV2 RNA is persistently detectable in the respiratory tract and in the intestinal epithelium several months after infection [32,33]. It is possible that the apparent increase in anti-S IgG persistence we have found is related to continued exposure to SARS-CoV-2 antigens long after a resolved infection. On the other hand, we cannot rule out subsequent unnoticed reinfections in the post-vaccination infected individuals. Importantly, since there is no well-defined humoral correlate of long-term protection [34], and both humoral and cellular immune mechanisms are important in the defence against infection, the anti-S IgG levels alone cannot be used to conclude on protection against SARS-CoV-2 infection or disease.

Several limitations of this study should be noted. The number of participants was small, female participants were overrepresented and the participants self-reported positive rapid antigen tests and vaccine types and dates (data was not cross-checked with the Norwegian Immunisation Registry). As the participants were trained medical personnel, we consider misreporting less likely. Nevertheless, as testing was initiated by the participants themselves, there is a chance that some asymptomatic infections went undetected, especially since the sensitivity of the anti-N assay used is generally low and decreases with time after infection [28]. The timing of the local pandemic peak gives a higher risk of unnoticed infections skewing data after the third dose. We did not analyse neutralising antibodies, which are considered to be the best humoral correlate of protection, but instead analysed antibodies binding the S1 or S2 domains of SARS-CoV-2 spike, including the receptor binding domain. Importantly, non-neutralising antibodies may be the first line of antigen-specific defence, mediating phagocytosis [35] and serving as surrogate markers of the T-cell response [36]. Moreover, a strong correlation has been observed between anti-S IgG titres and neutralising antibody titers [22,37]. Finally, the Liaison anti-S IgG assay used is not calibrated to the WHO International Standard (IS) for anti-SARS-CoV-2 immunoglobulin binding activity (NIBSC 20-136), and results are therefore not reported in binding antibody units per millilitre (BAU/mL), arguably making our results incomparable to other assays. However, given the enormous variation in reported antibody levels between calibrated assays from different manufacturers in head to head evaluations, the benefit of calibration is questionable [38], and the authors recommend an assay-specific approach.

One strength of this study is the long follow-up time of 21 months, which is considerably longer than most other COVID-19 vaccine studies.

## 5. Conclusions

In conclusion, we found that despite initial differences, after the third dose, all vaccine regimens elicited similar and high levels of anti-S IgG, which then declined to the same level over time, half a year later. The data presented here define a time- and dose-dependent anti-S IgG reference-level for the Liaison SARS-CoV-2 S1/S2 IgG assay. This together with the patient’s vaccination history, may facilitate interpretation of clinical anti-S IgG results. 

## Figures and Tables

**Table 1:** Description of participants.

	Pz-Short (n = 45)	Az-Pz (n = 21)	Pz-Long (n = 16)	Total (n = 82)
Age, years				
Mean (SD)	43 (11.3)	47 (13.2)	43 (12.4)	44 (11.9)
Median (Range)	45 (23–64)	51 (24–65)	47 (21–61)	46 (21–65)
Female, n	34 (76%)	20 (95%)	13 (81%)	67 (82%)
Vaccine				
1st dose, n	BNT162b2, 45 (100%)	ChAdOx1, 21 (100%)	BNT162b2, 16 (100%)	82 (100%)
2nd dose, n	BNT162b2 *, 45 (100%)	BNT162b2, 21 (100%)	BNT162b2, 16 (100%)	82 (100%)
3rd dose, n	BNT162b2/mRNA-1273, 37 (82%)	BNT162b2/mRNA-1273, 20 (95%)	BNT162b2/mRNA-1273, 15 (94%)	72 (88%)
3rd dose mRNA-1273, n	5/37	1/20	3/15	9/72
Days between 1–2 dose (mean)	21–26 (22)	68–90 (79)	28–42 (39)	
Days between 2–3 dose (mean)	205–337 (303)	196–314 (210)	149–380 (223)	

* One participant in this group received the mRNA1273 vaccine as the second dose.

**Table 2:** SARS-CoV-2 anti-S IgG concentration (AU/mL) post-vaccination.

Post Dose	Interval Days	Pz-Short	*p*-Value	Az-Pz	*p*-Value	Pz-Long	*p*-Value
1st	13–21	73 (55, 106); 21 (5)		23 (11, 32); 17 (1)		68 (47, 88); 11 (1)	
2nd	13–31	280 (239, 353); 39 (6)	<0.001	1160 (809, 1695); 19 (0)	<0.001	1075 (600, 1175); 16 (0)	<0.001
	80–120	214 (165, 266); 43 (0)	<0.001	310 (253, 380); 21 (0)	<0.001	233 (206, 260); 14 (0)	<0.001
	170–220	118 (90, 179); 42 (7)	<0.001	124 (108, 174); 17 (3/1)	<0.001	139 (116, 157); 13 (6)	<0.001
3rd	13–31	2920 (2220, 3120); 23 (1)	<0.001	2075 (1433, 3023); 16 (1)	<0.001	2390 (1993, 2763); 10 (0)	<0.001
	80–120	2325 (1403, 2803); 8 (0)	0.1325	1100 (923, 1350); 9 (0)	<0.01	1070 (963, 1255); 7 (0)	<0.05
	170–220	634 (341, 1121); 6 (0)	<0.01	306 (235, 353); 6 (0)	<0.05	918 (596, 1239); 2 (0)	0.8889
2nd	13–31	280 (239, 353); 39 (6)		1160 (809, 1695); 19 (0)		1075 (600, 1175); 16 (0)	
3rd	13–31	2920 (2220, 3120); 23 (1)	<0.001	2075 (1433, 3023); 16 (1)	<0.05	2390 (1993, 2763); 10 (0)	<0.001

Data shown are median (IQR); number of unique participants contributing to analysis, (number of participants with two/three samples in the same time interval). *p*-values from Wilcoxon two-sample tests comparing values of SARS-CoV-2 anti-S IgG concentration in the current time interval with the preceding time interval.

**Table 3:** Detection of SARS-CoV-2 infection. Data are n.

	Pz-Short	Az-Pz	Pz-Long	Total
Infection confirmed by PCR or antigen test	34/45 (76%)	17/21 (81%)	10/16 (63%)	61/82 (74%)
Analysed after confirmed infection	16/34 (47%)	13/17 (77%)	8/10 (80%)	37/61 (61%)
Two doses before infection	4	1	1	6
Three doses before infection	12	12	7	31
Anti-N IgG positive	10	11	6	27
Infection detected solely by anti-N IgG	3	0	2	5
Total infections detected	37/45 (82%)	17/21 (81%)	12/16 (75%)	66/82 (80%)

**Table 4:** SARS-CoV-2 anti-S IgG concentrations (AU/mL) post-vaccination and post-infection with combined vaccine groups.

	Interval Days	n	Mean [95% CI]	Fold Change	Slope [95% CI]	t-Value
Post 2nd dose	13–31	74 (80)	752 [661, 843]			
	80–120	78 (78)	306 [215, 397]	−2.5	−446 [−554, −338]	−8.112
	170–220	72 (90)	149 [61, 237]	−2.0	−157 [−262, −51]	−2.910
	13–220			−5.0		
Post 3rd dose	13–31	49 (51)	2496 [2257, 2739]			
	80–120	24 (24)	1440 [1135, 1741]	−1.7	−1057 [−1130, −774]	−7.518
	170–220	14 (14)	536 [173, 900]	−2.6	−903 [−1265, −544]	−4.915
	13–220			−4.6		
Post- infection	13–31	18 (19)	2760 [2340, 3172]			
	80–120	15 (15)	2040 [1589, 2493]	−1.4	−720 [−1287, −182]	−2.59
	170–220	15 (16)	2288 [1843, 2738]	+0.9	+248 [−324, 814]	0.852
	13–220			−1.2		

n: number of individuals with at least one observation per time interval (total number of observations). Mean, slope, t-value, and 95% CI from linear mixed model regression.

## Data Availability

Not applicable.

## Supplementary Materials

The following supporting information can be downloaded at: https://www.mdpi.com/article/10.3390/v15030619/s1, Figure S1: Histograms showing the distribution of sampling in the time intervals in the vaccine groups; Figure S2: Anti-S IgG (AU/mL) measured in individual participants over time, normalised at the second vaccine dose; Table S1: *p*-values; Table S2: Participants with serum samples after the third dose and distribution of mRNA-1273.
