# Supplementary material for: Dynamics of SARS-CoV-2 Spike-IgG throughout Three COVID-19 Vaccination Regimens: A 21-Month Longitudinal Study of 82 Norwegian Healthcare Workers
Source: Viruses. 2023 Feb 23;15(3):619. doi: 10.3390/v15030619 (PMC10056714; doi:10.3390/v15030619)
Supplement: Supplementary file 1 [file viruses-15-00619-s001.zip › Supplementary table 1 and 2.pdf]

Table S1. P-values

	P-values<br>Pz-short - Az-Pz	P-values<br>Pz-short - Pz-long	P-values<br>Az-Pz - Pz-long	P-values<br>Kruskal-Wallis chi-squared test all three groups
**1st vaccine dose**				
13-21 days	3.7e-5	0.6769	9.1e-4	1.4e-4
**2nd vaccine dose**				
13-21 days	6.1e-7	1.9e-5	0.3124	4.65e-8
80-120 days	1.0e-3	0.1976	0.07706	3.0e-3
170-220 days	0.2282	0.411	0.9333	0.4234
**3rd vaccine dose**				
13-21 days	0.1866	0.21	0.5512	0.2702
80-120 days	1.6e-2	0.09386	0.8371	4.3e-2
170-220 days	0.1797	0.8571	0.4286	0.2955

The Wilcoxon rank sum test together with the Kruskal-Wallis chi-squared test were used to compare antibody levels between groups within each time interval. Significant p-values are shown as exponential numbers.

Table S2. The number of participants with serum samples after the third dose, and the share receiving mRNA-1273

	Pz-short	Az-Pz	Pz-long	Total
**13-31 days**	23	16	10	49
**mRNA-1273**	3 (13%)	1 (6%)	2 (20%)	6 (12%)
**80-120 days**	8	9	7	16
**mRNA-1273**	1 (13%)	1 (11%)	1 (14%)	3 (19%)
**170-220 days**	6	6	2	14
**mRNA-1273**	0	1 (17%)	1 (50%)	2 (14%)
