# Supplementary figures and images for: Dynamics of SARS-CoV-2 Spike-IgG throughout Three COVID-19 Vaccination Regimens: A 21-Month Longitudinal Study of 82 Norwegian Healthcare Workers
Source: Viruses. 2023 Feb 23;15(3):619. doi: 10.3390/v15030619 (PMC10056714; doi:10.3390/v15030619)

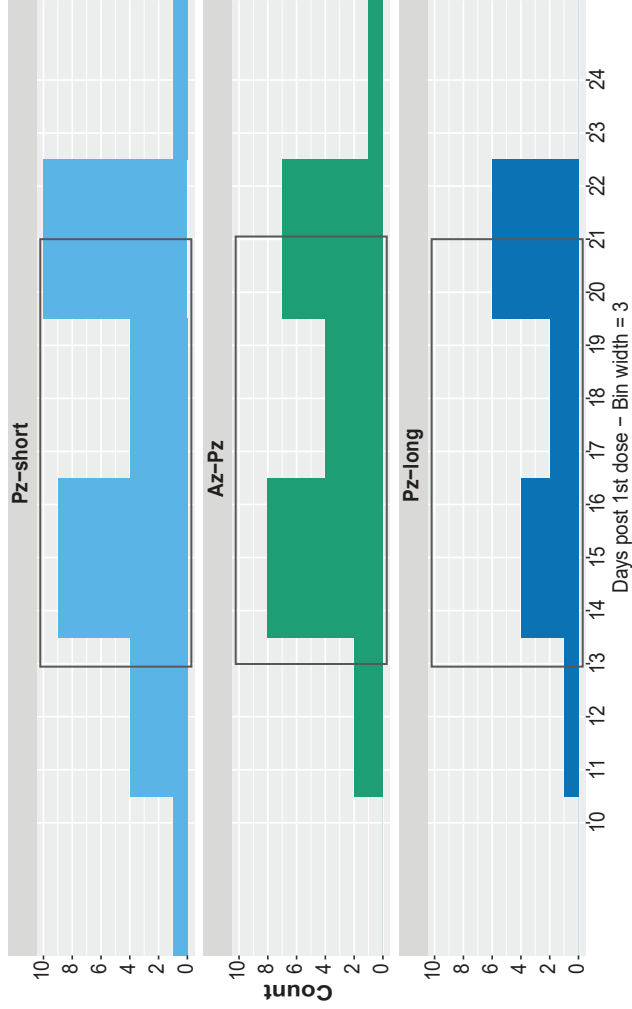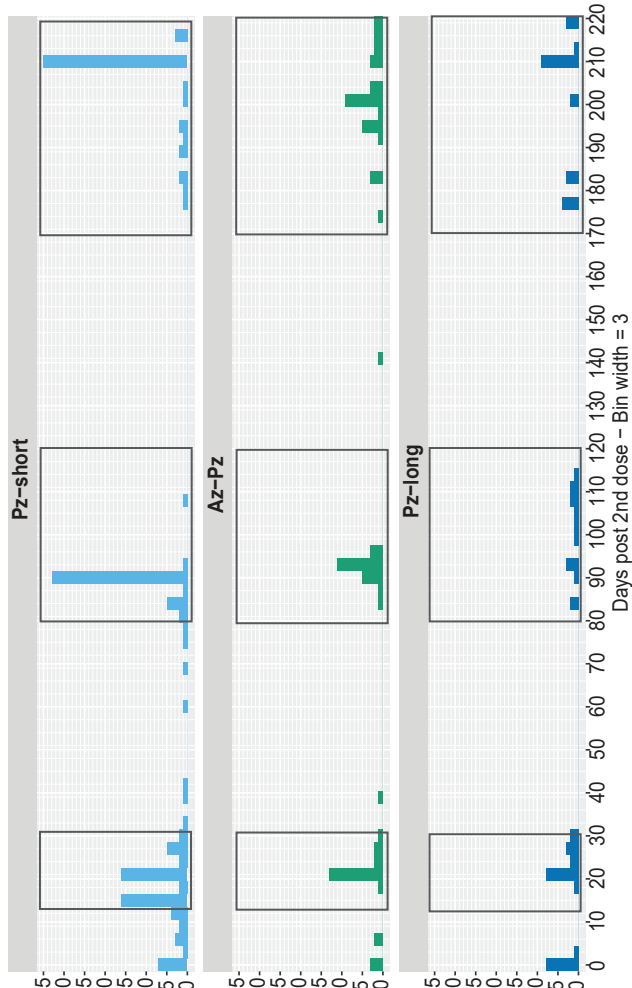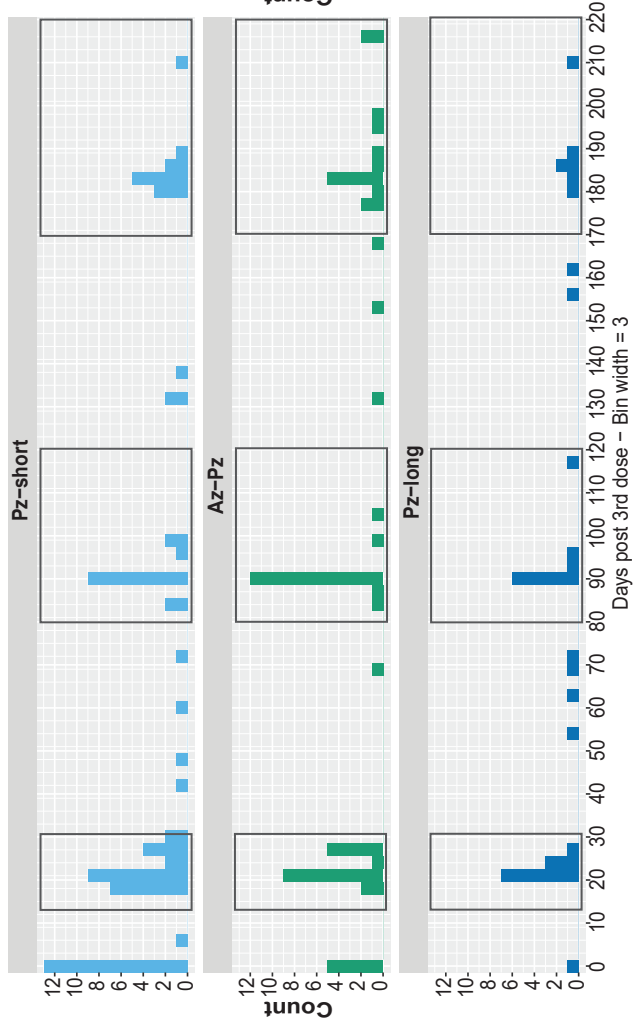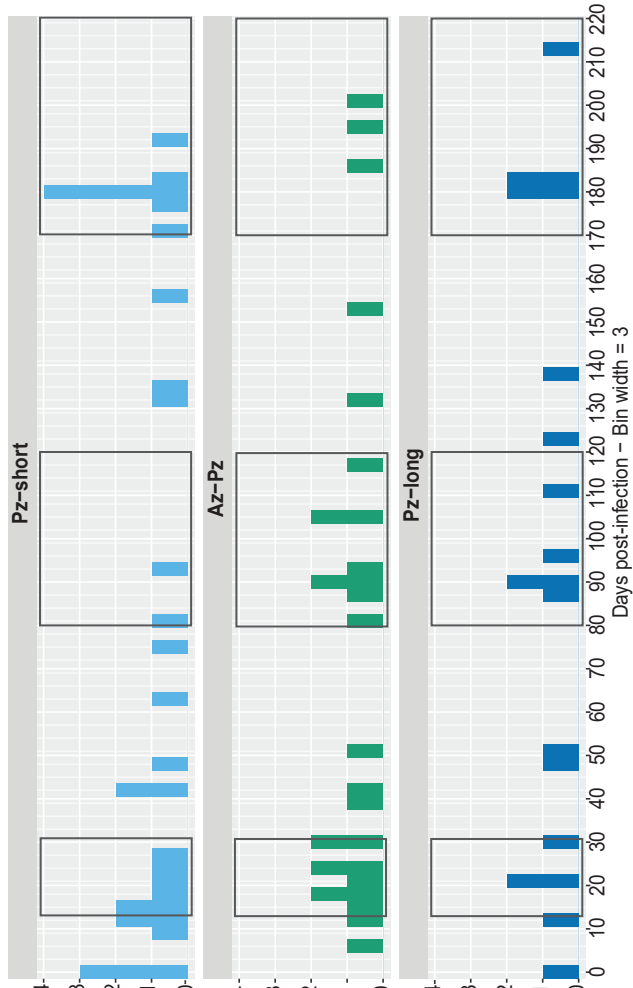

Supplement: Supplementary file 1 [file viruses-15-00619-s001.zip › Figure S1.pdf]

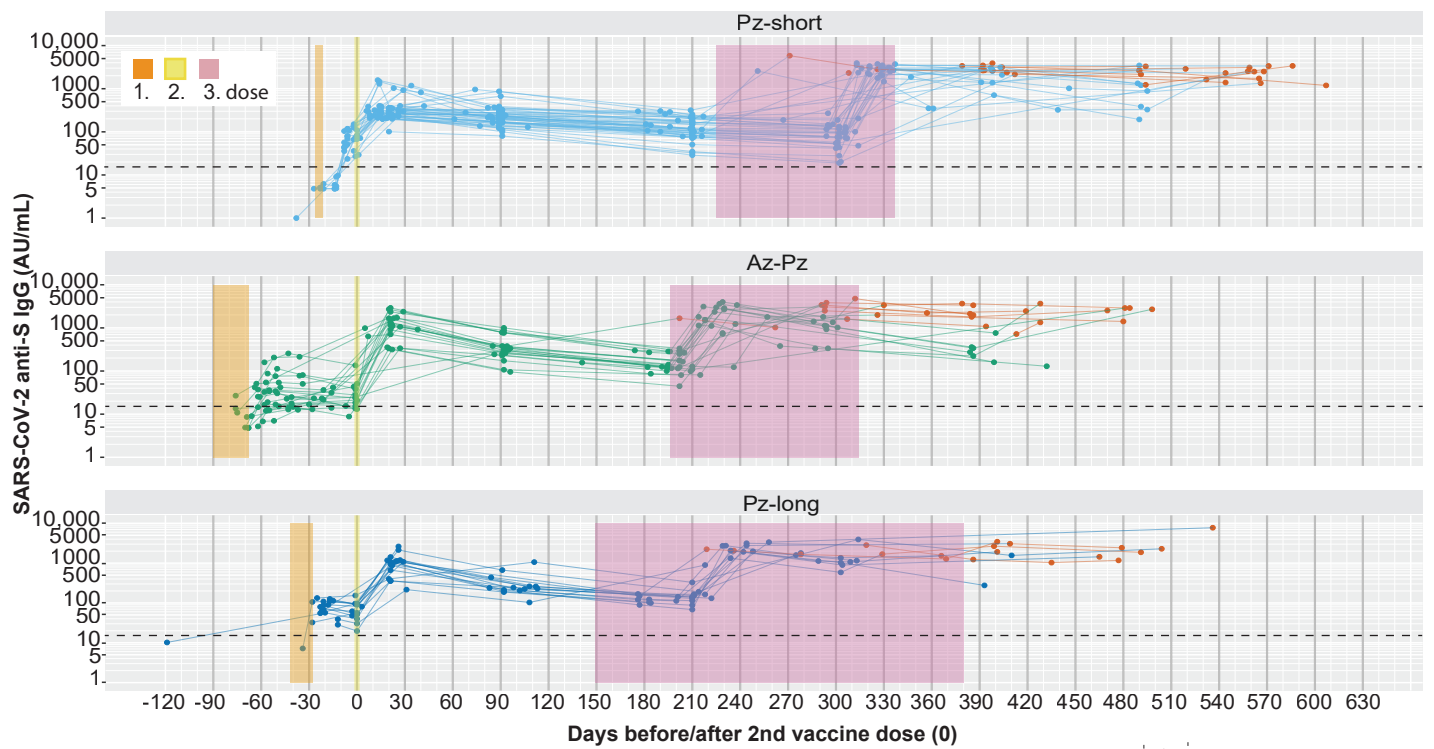

Supplement: Supplementary file 1 [file viruses-15-00619-s001.zip › Figure S2.pdf]
